# A deep learning-based automated Solar-Powered Fish Monitoring System

**DOI:** 10.1371/journal.pone.0351628

**Published:** 2026-06-18

**Authors:** Emmanuel Ahene, Richmond Owusu Agyei, Rose-Mary Owusuaa Mensah Gyening, Kwasi Adu Obirikorang, Kate Takyi, Kwabena Owusu-Agyemang, Linda Amoako-Banning, Joojo Walker

**Affiliations:** 1 Department of Computer Science, Kwame Nkrumah University of Science and Technology PMB, UPO, Kumasi, Ghana; 2 Department of Fisheries and Watershed Management, Kwame Nkrumah University of Science and Technology PMB, UPO, Kumasi, Ghana; 3 Chengdu University of Technology, Chengdu, China; Bangladesh Agricultural University, BANGLADESH

## Abstract

Green fish farming represents an integrated aquaculture approach that rears aquatic organisms in controlled environments to improve production efficiency and environmental sustainability. Although significant, current green fish farming practices are labour-intensive and expensive due to grid energy dependency resulting in operational inefficiencies and elevated fish mortality. To address these key challenges, we propose a multidisciplinary approach that involves the development of a cost-effective, solar-powered automation system that integrates computer vision and deep learning techniques for real-time monitoring of fish behaviour, water quality, feeding, and waste management. First, we design the system architecture that enables automation and ensures accurate system performance under varying conditions. Second, following the architecture, we build a complete and cost-effective smart system that works along with an intelligent software framework that leverages computer vision and deep learning techniques. Utilizing custom datasets from video frames and environmental sensors, this system utilizes convolutional neural networks (CNNs) for fish behavior analysis, real-time disease detection via camera feeds, and precise feeding control through actuators. The design also incorporates a renewable energy subsystem, employing advanced photovoltaic panels and efficient battery storage to guarantee reliable power. The major contribution lies in the seamless integration of these multidisciplinary components. Furthermore, the system architecture is modular and scalable, making it suitable for both smallholder and commercial fish farms. Cost optimization with low-cost sensors and open-source software enables economic viability for resource-constrained farmers. Extensive simulation studies confirmed significant improvements in monitoring accuracy, reduced manual intervention, and enhanced operational sustainability.

## Introduction

Recently, global food system faces unprecedented challenges in balancing increasing nutritional demands with environmental sustainability, particularly as climate change, population growth, and resource depletion strain conventional agricultural practices. In the face of these challenges, aquaculture has become a critical pillar of food security, supplying over 50% of the world’s seafood and serving as the fastest-growing food production sector over the past three decades [1]. In fact, acquaculture is expected to meet the world’s fish demand by 60% by 2030 [1]. However, the rapid expansion of traditional aquaculture has often come at significant ecological cost, including habitat degradation, water pollution, and overreliance on finite resources, prompting urgent calls for innovative, eco-conscious alternatives. Green fish farming stands out as one of the innovative fish farming alternatives that emphasizes energy efficiency, ecosystem preservation, and circular resource use. The green fish farming paradigm is expected to transform our approach to reconciling fish productivity with planetary health. Green fish farming enables the integration of renewable energy, innovative technologies, and waste-to-resource strategies to offer a pathway to sustainable food systems while addressing socioeconomic inequities in regions most vulnerable to food insecurity [2].

Fish and aquatic products constitute the primary source of animal protein for over 3 billion people worldwide. At the same time, the sector supports the incomes of approximately 20 million workers, predominantly in low- and middle-income countries. In nations such as Ghana, where over 60% of the population relies on fish for dietary protein and economic sustenance, aquaculture is not merely an industry but a lifeline. Yet, the sector remains constrained by persistent inefficiencies: manual labour dominates critical tasks like water quality monitoring, feeding, and waste management, while energy-intensive processes depend on erratic grid electricity or costly diesel generators [3]. These challenges exacerbate operational costs, elevate fish mortality rates, and restrict scalability, disproportionately affecting rural and coastal communities where poverty and resource limitations intersect.

While automation technologies hold promise for modernizing aquaculture, their adoption in resource-constrained settings remains negligible. Several developments have been made, given automation in the fish farming industry. In most cases, systems deployed for green fish farming require constant supervision, lack waste management support and rely on electrical power and backup generators from non-renewable sources [4]. Moreover, research has shown that most existing solutions are expensive [5]. These challenges inhibit their widespread adoption in coastal communities and rural areas and discourage fish farmers from adopting the sustainable green farming method.

To address these systemic barriers, we propose a multidisciplinary approach that involves the use of cost-effective components to build a low-cost, solar-powered automation system that integrates computer vision and deep learning techniques for real-time monitoring of fish behaviour, water quality, feeding, and waste management. Specifically, we make the following contributions:

1. First, we design the system architecture that enables automation and ensures accurate system performance under varying conditions.
2. Second, following the architecture, we build a complete solar-based smart system that works along with an intelligent software framework that leverages computer vision and deep learning techniques for fish behavior analysis, real-time disease detection via camera feeds, and precise feeding control through actuators.
3. We perform comprehensive experiment and case-based simulations to further confirm the significance of the proposed system following standard performance metrics.

Despite the potential benefits of automation, challenges such as dependency on inconsistent grid power, a lack of technical competence, and high maintenance costs usually delay technology adoption in developing countries such as Ghana. To address these issues, our design prioritizes low-cost, off-the-shelf components, modular scalability, and simple interfaces that require little user training. This strategy assures that the system stays practical, maintainable, and economically viable for smallholder fish farmers working in resource-constrained environments. The remainder of the paper is organized as follows; In the Related Works section, we present a comprehensive review of existing literature on green fish farming technologies. In the System requirements section, we define the requirements of the proposed system. In the Materials and Methods section, we describe the various components, materials and the deep learning techniques used in building the low-cost automated green fish farming system. In the Experiment and Simulation section, we present the complete system and discuss the results obtained from our comprehensive experiment in the Results and Discussions section. The Conclusion section, presents the conclusion of the paper.

## Related works

This section provides a comprehensive review of existing literature on green fish farming, automation in aquaculture, and renewable energy solutions. It examines previous research on automated fish feeding systems, the integration of computer vision technology in aquaculture, and the role of solar energy in sustainable fish farming. Additionally, we analyze the challenges and limitations identified in prior studies to highlight the gaps that the proposed system addresses. Dinh et al. [6] explored the application of Internet of Things (IoT) technology in modernizing fish farming practices through the development of a water quality monitoring and automatic correction system. The system monitors four key parameters: temperature, pH level, turbidity, and dissolved oxygen, using a combination of sensors, microcontrollers, and a mobile application for real-time data acquisition and monitoring. Notifications are sent to users whenever any parameter deviates from standard values. An Arduino Nano is employed as the control unit to read analog sensor data, while a serverless IoT platform is implemented using Firebase Realtime Database (RTDB) and ESP8266. The system was experimentally validated at the Faculty of Fisheries Technology and Aquatic Resources, Maejo University, demonstrating its potential for enhancing automation and efficiency in aquaculture operations.

Hossam et al. [7] presented an innovative system integrating computer vision and IoT technologies to optimize feeding in tilapia aquaculture. Real-time IoT sensors monitor water quality parameters, while computer vision algorithms analyze fish size and count to determine precise feeding quantities. A mobile application enables remote monitoring and control. The system employs YOLOv8 for keypoint detection to estimate fish weight from length, achieving 94% precision on 3,500 annotated images. Pixel-based measurements are converted to centimeters using depth estimation, enhancing feeding accuracy. By aligning data collection with inference conditions, the method significantly improved performance. Preliminary results indicate that this approach could boost production up to 58 times compared to traditional farming methods. All models, code, and datasets are made open-source [8].

A study by Naphade et al. [9] proposed an IoT-based intelligent monitoring and maintenance system to support aquaculture farming, particularly in the context of pearl farming, aimed at helping farmers adapt to challenges posed by climate-induced agricultural losses. The system monitors water quality and forecasts changes in key water parameters using an ensemble learning approach based on random forests (RF). Performance comparisons with linear regression (LR), support vector regression (SVR), and gradient boosting machine (GBM) models showed that the RF model achieved superior forecasting accuracy, with a mean absolute error (MAE) of 1.428 for dissolved oxygen (DO) and 0.141 for pH prediction. A study by Das et al. [10] presented an IoT-based water quality monitoring system for aquaculture, leveraging Raspberry Pi, Arduino, various sensors, a smartphone camera, and an Android application. The system measures key water parameters including temperature, pH, electrical conductivity, and color. Sensor data is acquired via Arduino, processed and stored using Raspberry Pi, while water color is detected through photo acquisition. The Android app enables users to monitor water conditions locally over Wi-Fi and remotely via the Internet. Basic analysis of parameter values provides users with actionable insights into the overall water quality, enhancing aquaculture management. Tolentino et al. [11] presented a water quality monitoring and automatic correction system for intensive aquaculture setups, aiming to optimize fish growth and reduce fish kills. The system monitors six vital parameters: temperature, pH, oxidation reduction potential, turbidity, salinity, and dissolved oxygen, using Arduino, Raspberry Pi 3B + , and the LoRaWAN IoT protocol. Automated corrective devices, such as heaters, sodium bicarbonate dispensers, solenoid valves, and water pumps, maintain optimal conditions. Comparative experiments between controlled and conventional aquaculture setups demonstrated that the controlled system significantly improved efficiency, reduced labor, prevented fish kills, and enhanced yield quality.

Quaade et al. [12] explored the use of computer vision and remote sensing imagery to improve the monitoring of aquaculture production. Their methodology involved training a computer vision model to detect marine aquaculture cages from aerial and satellite imagery, creating a spatially explicit dataset of finfish production locations in the French Mediterranean from 2000 to 2021. The study highlighted the efficiency, scalability, and adaptability of this approach, which offers a cost-effective solution for aquaculture surveys. Their findings demonstrated that this method can enhance the speed and reliability of data collection and can be used to estimate production levels and quantify uncertainty in these estimates. This research presents a valuable tool for researchers and regulators involved in aquaculture monitoring and management.

Smith et al. [13] developed a low-cost computer vision system to analyze fish behavior in aquaculture tanks, aiming to quantify behavioral variations that are typically challenging to measure. Their system enables the simultaneous observation of nine tanks, allowing the study of a single factor across three triplets for statistical analysis. The use of a web publishing tool provides continuous monitoring, while remote control capabilities ensure that human presence does not introduce behavioral variations. The system was evaluated by measuring fish interactions, such as inspection and biting, under three different net conditions, with stocking density used as a stress factor. The results demonstrated that the system successfully recorded fish behavior with minimal frame loss (<21 s in 24 h) and accurately identified every fish interaction with the net. Additionally, the study found no statistical differences in fish behavior variations within a single day. This research presents an affordable and efficient solution for monitoring and analyzing fish behavior in aquaculture settings.

Zhour et al. [14] designed an intelligent feeding controller for aquaculture based on machine vision and feeding behavior analysis to address the limitations of existing feeding devices. The hardware platform was built on the I.MX6 microcontroller, and the software was developed using embedded Linux OS. The system utilized image processing to analyze fish feeding behavior and control the feeding process automatically. By collecting and analyzing images of the feeding process, the authors applied Delaunay Triangulation to extract the flocking index of fish feeding behavior (FIFFB). Feeding decisions were made based on a defined threshold to adjust the amount of feed according to the fish’s appetite. The results demonstrated that the proposed system is more intelligent than traditional feeders, reducing feed waste and water pollution. This automated control improves feeding efficiency and supports sustainable aquaculture practices.

Lim et al. [15] explored the implementation of artificial intelligence (AI) in aquaculture and fisheries, focusing on deep learning, machine vision, big data, the Internet of Things (IoT), and robotics. In his article, he discussed how these technologies are revolutionizing the industry by enabling more efficient and sustainable practices. The integration of AI offers solutions to major challenges such as feed optimization, water quality monitoring, and automated fish behavior analysis, all of which contribute to improved productivity and reduced environmental impact. Lim’s work highlights the potential of these advanced technologies to drive the future of aquaculture and fisheries, ensuring a more sustainable and high-throughput industry.

Zion et al. [16] developed an image-processing algorithm to successfully discriminate among three fish species—common carp (Cyprinus carpio), St. Peter’s fish (Oreochromis sp.), and grey mullet (Mugil cephalus)—based on side-view images captured while the fish were swimming in an aquarium. The algorithm utilized moment-invariants (MI) combined with geometrical considerations, making it insensitive to fish size, two-dimensional orientation, and location within the camera’s field of view. A total of 143 images were collected and divided into two sets for a two-fold cross-validation test. The model achieved species identification accuracies of 100% for grey mullet, and 91% for both carp and St. Peter’s fish. This study represents the first reported success in in vivo discrimination among fish species using image processing and further explored the feasibility of training fish to swim through a narrow Plexiglas channel, offering prospects for automated sorting systems.

Zhou et al. [17] developed an automatic method for grading fish feeding intensity using a convolutional neural network (CNN) and machine vision to address the inefficiencies and subjectivity of traditional fish appetite assessment methods in aquaculture. Their approach involved collecting images during the feeding process, constructing a dataset expanded through rotation, scale, translation (RST) augmentation techniques, and noise-invariant data expansion. A CNN model was trained on this dataset to classify fish appetite levels automatically. Upon evaluation, the method achieved a grading accuracy of 90%, demonstrating its potential for guiding feeding and production practices through reliable and efficient fish appetite detection.

Alselek et al. [18] examined the development of an innovative aquaponics health monitoring system to address the growing demand for sustainable fish farming. The study integrated high-tech sensors to monitor fish and crop health while employing a data analytics framework to provide actionable feedback to farmers. It highlighted advancements in life cycle monitoring metrics and communication technologies while incorporating energy-efficient solutions to enhance sustainability. The findings demonstrate how technological innovations can optimize productivity and support scalable aquaponics farming practices. Ozigbo et al. [19] focused on the development of an automatic fish feeder designed to optimize feeding processes in aquaculture systems. The research highlights the use of automated mechanisms to ensure precise feeding schedules and quantities, which reduces manual labor and minimizes feed wastage. The system leverages sensors and timers to enhance efficiency and ensure consistent feeding practices, contributing to improved fish growth and health. The findings underscore the potential of automation in aquaculture to improve productivity and sustainability. Ullo et al. [20] explored the advancements in smart environments. This study explores methods and strategies for integrating sensors to automate labor-intensive processes in fish farming. By integrating IoT and sensor technologies, their paper sought to enhance efficiency, improve outcomes and reduce the cost of widespread adoption for local fish farmers in rural and coastal areas. The research highlights the role of integrated IoT frameworks in efficiently collecting and analyzing environmental data, ensuring sustainability and protection. It emphasized the significance of employing advanced sensor networks for real-time monitoring, automated control, and improved decision-making processes. The findings underline how IoT-driven solutions are transforming environmental monitoring by addressing challenges like scalability, energy efficiency, and data accuracy to enhance overall system performance. This research by Olanubi et al [21] focused on the development of an IoT-based intelligent water quality management system designed to optimize aquaculture monitoring. The study highlighted the use of automated sensors to continuously track key water parameters like temperature, pH, and turbidity, ensuring a stable aquatic environment. The system used an ESP32 microcontroller to collect and transmit real-time data to a cloud database, enabling farmers to remotely monitor and control water quality through a web application. Additionally, automated alerts and notifications via WhatsApp Messenger enhanced responsiveness, reducing the risks associated with poor water conditions. The findings underscored the potential of automation in aquaculture to improve productivity and sustainability by ensuring efficient water quality management and reducing manual monitoring efforts. Alver et al. [22] developed a device based on computer vision to accurately quantify the feed density within a specified volume of a sea cage in marine fish farming. Their methodology involved designing a physical device and applying a combination of well-established algorithms to detect and quantify feed pellets reliably. The findings showed that the device achieved detection and quantification with an error of 1.3%, addressing the scarcity of proper validation data and supporting optimized fish growth and welfare through improved feed distribution modeling.

A study by Lindholm-Lehto [23] focused on the importance of water quality monitoring in recirculating aquaculture systems (RAS) and emphasized the role of the Internet of Things (IoT) and artificial intelligence (AI) in providing real-time water quality assessment. The study gave an overview of the various sensors and monitoring methods utilized to measure important water quality metrics. Traditional approaches rely on handheld sensors and laboratory studies, but modern IoT-based solutions provide continuous monitoring and automated alerts. They also examined the limitations of current monitoring systems, such as the requirement for skilled users, frequent maintenance, and calibration. The study also investigated how changes in fish behavior, such as swimming activity and acceleration, can serve as markers of water quality. Awais et al [24] explored the creation of an AI-powered automated irrigation system aimed at optimizing solar energy utilization for agricultural irrigation. The study focused on the application of machine learning models, such as a spatial and temporal attention block-based long short-term memory (LSTM) network, to forecast high-efficiency irrigation system power production. The system provided sustainability and efficiency by generating solar photovoltaic power, while also addressing problems related to weather variability and technical feasibility. The results showed that the proposed model outperforms existing machine learning and LSTM-based models, improving Mean Absolute Percentage Error (MAPE) by 6–7% via improved look-back and look-forward processes [25]. A proposal on future enhancements to adapt the technique for wind power production and increase forecasting accuracy based on consumer behavior was made. Their findings highlighted the potential for AI-driven automation in solar irrigation, which can contribute to more sustainable and ecologically friendly farming practices.

Ubina et al. [26] developed a two-stage approach to improve fish feeding intensity evaluation for aquaculture farming. Their methodology involved applying an optical flow neural network to generate optical flow frames, which were then fed into a 3D convolutional neural network (3D CNN) for intensity evaluation. Using aerial drone footage, they captured RGB water surface images during fish feeding activities, processed them with deep optical flow models, and manually annotated feeding levels into four categories: ’none,’ ’weak,’ ’medium,’ and ’strong.’ Their findings showed that the proposed method achieved up to 95% accuracy, outperforming existing CNN-based systems for evaluating fish feeding intensity.

Priya et al [27]. investigated the development of an IoT-based power monitoring system with edge intelligence to improve energy management, reliability, and sustainability in smart solar arrays and substations. The study described a high-accuracy power predicted system (98%), which decreased power fluctuations by 30% and energy management expenses by 95%. The system supported real-time monitoring and decision-making, with a response time of less than one second. It increased power distribution efficiency by 25% and decreased downtime by 40% in a variety of commercial, residential, and industrial applications. By combining IoT with edge intelligence, the framework improved safety, sustainability, and energy management in smart buildings by up to 30%. The results show how real-time IoT analytics may be used in Industry 4.0 applications to maximize energy efficiency, power generation, and distribution. Krishna et al. [28] developed an IoT-based framework to optimize solar power efficiency through advanced real-time monitoring and smart system integration. The study explored IoT’s role in improving solar energy management, optimizing resource utilization, and enhancing system reliability. The findings highlight that leveraging IoT technologies can streamline solar power operations while reducing costs and improving response times. This research underscores the value of integrating IoT solutions for efficient renewable energy use. Abdulmouti et al. [29] explored the feasibility of integrating solar-powered systems into aquaponics for sustainable agriculture. The study evaluated how solar energy can support aquaponics systems, focusing on resource management, energy reliability, and environmental sustainability. Findings suggested that solar-powered aquaponics can enhance operational efficiency, minimize costs, and promote sustainable food production by addressing energy consumption challenges. This research highlighted the viability of renewable energy in supporting innovative agricultural practices. Sujathakumari et al. [30] proposed an eco-friendly solar-powered irrigation system that will help farmers better control their water resources and use less energy. The study emphasized the use of solar energy and smart irrigation to promote sustainability and crop output. The findings showed that using solar technology can greatly improve operating efficiency while lowering dependency on traditional energy sources. This study highlighted the importance of smart irrigation systems in promoting sustainable and innovative agriculture practices.

Shitsukane et al. [31] explored the integration of fuzzy logic, solar power, and IoT technologies to optimize energy management and environmental monitoring systems. Their research focused on applying these technologies to improve sustainability, system efficiency, and resource allocation. Findings suggested that combining fuzzy logic with solar-powered IoT systems enhances real-time decision-making processes and supports eco-friendly applications in smart environments. This work highlighted the potential of interdisciplinary technological solutions for addressing environmental and energy challenges. Abdallat et al. [32] explored the development of a sustainable, green, and filtration-based system integrating renewable energy solutions to address environmental challenges. The study focused on combining renewable energy technologies with filtration processes for water treatment and purification. Their findings highlighted the potential of green systems in reducing environmental degradation while ensuring a clean water supply. The study emphasized system design optimization and practical applications for sustainable and renewable energy use in filtration systems. Gaspar et al. [33] examined the development of a low-cost, solar-powered, modular aquaponics system. Their study focused on leveraging solar energy and sustainable design to create efficient aquaponics solutions suitable for small-scale farming. They highlighted how this system integrates renewable energy with aquaponic technology to improve resource management and agricultural productivity. The findings demonstrated its potential as a cost-effective, scalable, and environmentally sustainable solution for modern aquaponics applications.

While existing IoT frameworks in aquaculture ([18,34,35]) demonstrate robust capabilities for real-time data collection (water quality, temperature), they mainly focus on passive monitoring rather than predictive or proactive solutions. For instance, Ali et al. [18] and Aishwarya et al. [34] rely on cloud-based analytics, which introduce latency and energy costs, while Ozigbo et al. [19]’s automated feeder lacks adaptive scheduling based on environmental conditions. Most importantly, none integrate machine learning to forecast critical events such as dissolved oxygen depletion and disease outbreaks or address energy constraints in off-grid deployments, as highlighted in Abdulmouti et al. [36]’s solar feasibility study. The proposed solution bridges these gaps by deploying low-power IoT sensors with edge-based AI models to enable localized, real-time decision-making, such as remotely controlling oxygen pumps or feeders preemptively. Furthermore, by incorporating solar-hybrid energy modules inspired by Gaspar et al. [33], our system ensures sustained operation in resource-limited settings, achieving a cost reduction compared to conventional setups. This integration of edge intelligence and energy autonomy directly addresses scalability and accessibility gaps overlooked in prior works ([18,20,35]). The proposed approach, therefore, offers a holistic, scalable solution designed for the needs of low-income regions, where aquaculture directly supports food security and economic resilience.

### System requirements

This study aims to design a solar-powered automated fish farming system that reduces labour and mortality rates through IoT-enabled water monitoring (pH, temperature, dissolved oxygen), AI-driven predictive control using adaptive feeding schedules, and waste management. By integrating solar energy with long-lasting energy storage batteries as backup and smart load prioritization, the system eliminates grid dependency while ensuring longer operation time for critical functions such as aeration in water. A farmer-friendly Android and web app provides real-time alerts and remote control, and has been validated through usability testing with local farmers. The system is designed for easy scalability and affordability for small-scale operations.

The system requirements are to: (i) monitor the various parameters of water such as pH level, temperature, water level, and dissolved oxygen level, (ii) constantly supply enough oxygen to the fish in the tank, (iii) monitor the health of fish and automate the feeding process, (iv)supply stable and reliable power without relying on external energy sources, (v) manage waste produced by fish, (vi) create a user-friendly mobile and web app for local fish farmers, (vii)assemble a low-cost, solar-powered automated fish farming equipment, (viii)promote sustainable and environmentally friendly fish farming practices.

## Materials and methods

### IoT integration

**Fig 1:**
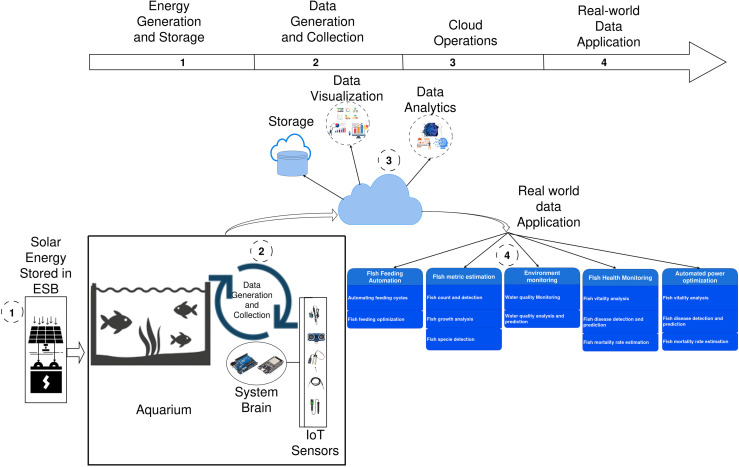
Architecture of the proposed system.

Fig 1 shows the architectural overview of our proposed system. The proposed system integrates IoT-enabled sensors, solar energy autonomy, and edge-based automation to address labour-intensive and energy-dependent challenges in small-scale aquaculture. The architecture utilizes an ESP32 microcontroller interfaced with a number of sensors: temperature sensor (1-Wire protocol, GPIO 4), analog pH sensor (ADC, GPIO 34), and ultrasonic water-level sensor to continuously monitor water quality parameters such as temperature, acidity, and dissolved oxygen. Sensor data is transmitted via Wi-Fi to a PostgreSQL cloud database for real-time visualization on Grafana dashboards, while the ESP32 executes edge-based logic to trigger the actuators (aerator pumps via relays) when thresholds are exceeded. This localized decision-making ensures responsiveness even during connectivity disruptions.

To maintain the system’s accuracy, we regularly calibrate the pH sensor using standard buffer solutions (pH 4.0(acidic), 7.0(neutral), and 10.0(basic/alkaline)). Additionally, we conducted extended testing in low-light conditions to assess its performance over time. Security is enforced via TLS encryption for data transmission. Automation of feeding is achieved through a servo-driven dispenser with precision-drilled holes, releasing controlled feed quantities at 2-hour intervals. The feeding system automatically adjusts based on the fish’s behavior, which is monitored through a camera. When fish become less active, the speed of the servo motor is gradually slowed down to reduce the amount of feed dispensed. The servo motor completely shuts down by resetting its angle to zero degrees once the fish are full. This is to prevent waste and ensure they are only fed when needed. Concurrently, an aquarium filter removes residual feed and organic waste, maintaining water quality. Energy autonomy is ensured by a solar-hybrid system comprising a 20W solar panel, two parallel 12V 5Ah LiFePO4 batteries, and a PWM charge controller. The Raspberry Pi, powered via a 5V DC power supply, gathers sensor data and hosts a farmer-friendly Android or web app for remote monitoring, while the ESP32 operates in deep sleep mode to reduce idle power consumption by 80%. A 12V inverter supplies AC power to high-load components (aerators, aquarium filter), with smart load prioritization redirecting energy to critical systems during low-battery conditions. Voltage thresholds (11.5V cutoff) and dual-battery redundancy prevent unplanned system shutdown.

### User-centric mobile/Web app and disease detection framework

**Fig 2:**
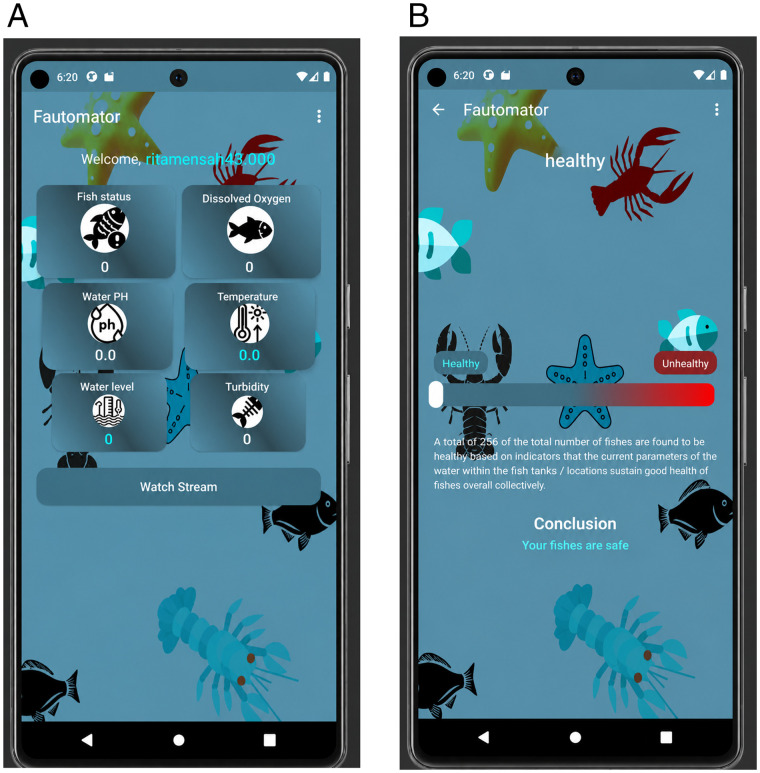
Sensor streaming page and AI support interface. (a) Streaming interface (b) AI support page.

The system incorporates a user-friendly mobile and web application that enables farmers to monitor and control aquaculture operations remotely as shown in Fig 2. Users authenticate via secure login credentials, with new users guided through account creation interfaces. Upon login, the dashboard displays real-time sensor values (dissolved oxygen, pH) and detection results through clickable widgets as shown in Fig 2a. For instance, clicking the temperature widget reveals contextual insights, such as ideal ranges for target fish species. A dedicated video-streaming interface, accessible via an on-screen button, provides live footage of fish behaviour overlaid with AI-driven disease detections as shown in Fig 2b.

To enable fish and disease detection, the system integrates a fine-tuned YOLOv5 [37] model trained on a blend of labeled fish disease dataset sourced from Roboflow [38] with frames of healthy fish extracted from videos downloaded from Pexels and Youtube. In total, the dataset consists of 876 images with 303 images of healthy fish and 573 images of unhealthy fish, which we used to train the model. We acknowledge that the number of images used for training may not be sufficient to ensure strong generalization across diverse real-world conditions.

We chose YOLOv5 for its ease of fine-tuning, ability to run on limited resources, and superior performance in object detection tasks compared to other models. Each image was annotated with bounding boxes classifying fish health into five categories: healthy, parasitic disease, saprolegniasis, bacterial gill disease, and white tail disease. The model achieved high confidence rates in real-time inference, detecting diseases directly from the live video feed. Detections are displayed as bounding boxes with labels and confidence scores, alerting users to act promptly, for example, isolating infected fish or adjusting oxygen levels via the app’s manual controls. Farmers can override automated systems through the app, such as triggering supplemental feeding or activating aerators, while the cloud server accounts for all system functionalities. The integration of YOLOv5’s precision with intuitive app design bridges the gap between advanced AI diagnostics and practical usability for non-technical users, ensuring scalable adoption.

### Ethics statement

Informed verbal consent was obtained from all participating farmers before collecting feedback on the application design. Participants were informed about the purpose of the study, the voluntary nature of participation, and the intended use of their feedback. The verbal consent process was documented by the research team through written notes and was witnessed by a member of the research team.

### Data preprocessing

The dataset consists of over 300 images of healthy fish. All images were resized to 320x320 pixels using the OpenCV library in Python to ensure consistency and reduce computational overhead during model training. In addition to the dataset are 573 inclusive images of unhealthy fish. We recognize that the amount of images utilized in training may be insufficient to provide high generalization across varied real-world settings.

**Data Augmentation:** Various image augmentation techniques were applied to improve model generalization and simulate real-world variations such as: (i) Random Rotations: Within ± 30 degrees to simulate different orientations of fish. (ii)Cropping and Scaling: To handle occlusion issues. (iii)Horizontal and Vertical Flipping: This accounts for symmetry in fish orientations.

***Data Splitting*:** The dataset was partitioned into two sets to facilitate effective training and testing. (i) Training (70%): Used for training the model (ii) Validation (20%): Used to evaluate model performance during training. (iii) Testing(10%): Reserved for the final evaluation of the trained model. In this paper, YOLOv5 was selected due to its efficiency, ease of use, and strong object detection capabilities, offering a balance between speed and accuracy. Its customizability allows for easy fine-tuning to our fish detection task. Specifically, we used the YOLOv5s variant [38], as it provides an optimal trade-off between processing efficiency and detection accuracy, making it ideal for deployment on resource-constrained devices like the Raspberry Pi 4 used in our project.

Training was performed using Jupyter Notebook, an open-source interactive computing environment. The implementation was done in Python 3.8, using the Ultralytics library for YOLOv5 and PyTorch as the deep learning framework. YOLOv5s pre-trained model was used as a starting point for the training on the dataset(Roboflow [38]). To ensure consistency in image shapes, they were resized to 320x320 pixels. The batch size was set to 16, and the total epochs were 50.

The performance of the model after training was assessed using evaluation metrics. These metrics helped to better understand the model’s accuracy, precision, and overall effectiveness in various conditions. By using these metrics, the performance of the model was frequently assessed and optimized, ensuring it meets the requirements for accuracy and robustness in real-world applications. The main evaluation metrics used are:

***Accuracy:*** The ratio of correctly predicted instances(both true positives and true negatives) to the total instances.


$$\begin{array}{c} Accuracy=\frac{\text{TP}+\text{TN}}{TP+TN+FP+FN} \end{array}$$ (1)

***Precision*:** The ratio of true positive detections to the total number of positive detections(true positives and false positives)


$$\begin{array}{c} Precision=\frac{TP}{TP+FP} \end{array}$$ (2)

***Recall(Sensitivity):*** The ratio of true positive detections to the total number of actual positives (true positives and false negatives)


$$\begin{array}{c} Recall=\frac{TP}{TP+FN} \end{array}$$ (3)

***F1-Score*:** The harmonic mean of precision and recall provides a balance between the two.


$$\begin{array}{c} F1Score=2\times \frac{Precision*Recall}{Precision+Recall} \end{array}$$ (4)

***Mean average Precision(mAP)*:** Measures the precision and recall performance of a model across multiple queries or classes.


$$\begin{array}{c} mAP=\frac{1}{n}\sum_{i=1}^{n}AP_{i} \end{array}$$ (5)

Where n is the number of classes and AP is the average precision of the classes.

### System configuration and mathematical modelind

Case 1: PV Integrated with Energy Storage In this scenario, the photovoltaic (PV) system is integrated with an energy storage battery (ESB), allowing the generated electricity to be stored and later used to power local loads. The output power of the solar panels depends on two key factors: solar irradiance (G) and temperature (T). The relationship can be expressed mathematically as follows [39,40]:


$$\begin{array}{c} P_{\text{pv}}=G\cdot A\cdot \eta \cdot (1-\gamma )\cdot (T-T_{\text{ref}}) \end{array}$$ (6)

where $P_{pv}$ is the photovoltaic (PV) system output power, A is the total area of the solar panels, η is the efficiency of the solar panels, γ is the temperature coefficient, T is the actual temperature, and $T_{ref}$ is the reference temperature.

Case 2: Solar panel + with ESB, we have a PV system that is connected to a set of storage batteries. Current is generated from the solar panels and stored in the ESB. Battery state of charge (SoC) This defines the amount of energy stored in the batteries at a given time. It is expressed mathematically as [41,42]:


$$\begin{array}{c} SoC=(E_{n}-E_{min})/(E_{max}-E_{max}) \end{array}$$ (7)

where SoC is the battery state of charge, $E_{n}$ is the energy stored in the batteries, $E_{min}$ is the minimum allowable energy level, and $E_{max}$ is the maximum energy storage capacity. Battery charging and discharging: The flow of power into and out of the batteries depends on the SoC and the charging or discharging efficiency. The charging and discharging power can be represented as [43]:


$$\begin{array}{c} P_{charge}=\eta_{\text{charge}}\cdot P_{\text{pv}}-P_{\text{load}} \end{array}$$ (8)

$$\begin{array}{c} P_{discharge}=\eta_{\text{discharge}}\cdot P_{\text{load}}-P_{\text{pv}} \end{array}$$ (9)

where $P_{charge}$ is the power flow into the batteries, $P_{discharge}$ is the power flow out of the batteries, $\eta_{charge}$ is the charging efficiency, $\eta_{discharge}$ is the discharging efficiency, $P_{pv}$ is the PV system output power, and $P_{load}$ is the local load demand.

### Energy cost(EC)

To improve self-consumption and self-sufficiency in non-grid-reliant systems through the use of energy storage batteries integrated with solar panels, a cost of energy model can be developed. Such a model helps to analyze the economic viability of the system. The cost of energy model equations is expressed in terms of:

1. The cost of electricity based on the system components is the average cost of generating electricity(CE) over the lifetime of the system, including installation costs, operation, and maintenance. The $CE_{s}$ can be computed using the formula:


$$\begin{array}{c} CE_{s}=(CT+COM)/(EG\cdot LG) \end{array}$$ (10)

where, $CT_{s}$ is the sum of the Solar system cost and the ESB cost, COM is the annual operation and maintenance costs, EG is the annual electricity generation from the solar system, and LG is the system life-time in years.

### Battery charging rate and efficiency modeling

The performance of the battery storage system is characterized by the instantaneous charging current, C-rate, and overall efficiency. These parameters depend on the photovoltaic (PV) power generation, charge controller efficiency, and the energy conversion losses in the battery. The following equations describe the relationships used in the analysis. The power generated by the PV panel under a given solar irradiance *G* is given as: [39,40]:


$$\begin{array}{c} P_{\text{pv}}(G)=P_{\max}\cdot \frac{G}{1000} \end{array}$$ (11)

where $P_{\max}$ is the rated peak power of the solar module at standard test conditions (STC: 1000 *W*/*m*^2^, $25^{\circ }C$).

The available charging power after accounting for charge controller efficiency is expressed as:


$$\begin{array}{c} P_{\text{avail}}=\eta_{\text{ctrl}}\cdot P_{\text{pv}} \end{array}$$ (12)

where $\eta_{\text{ctrl}}$ is the charge controller (or MPPT) efficiency.

The instantaneous battery charging current is then computed as:


$$\begin{array}{c} I_{\text{charge}}=\frac{P_{\text{avail}}}{V_{\text{batt}}} \end{array}$$ (13)

where *V*_batt_ is the measured battery voltage.

The corresponding C-rate, representing the normalized rate of charging with respect to the rated capacity of the battery, is defined as:


$$\begin{array}{c} C_{\text{rate}}=\frac{I_{\text{charge}}}{C_{\text{batt}}} \end{array}$$ (14)

where *C*_batt_ is the battery capacity in ampere-hours (Ah).

The actual rate of energy stored in the battery, considering the charging efficiency of the battery, is given as:


$$\begin{array}{c} E_{\text{stored\_rate}}=\eta_{\text{batt}}\cdot P_{\text{avail}} \end{array}$$ (15)

where $\eta_{\text{batt}}$ represents the efficiency of the battery’s electrochemical charging process.

Finally, the fraction of PV power successfully stored in the battery system is given by:


$$\begin{array}{cc} \text{Fraction of PV power stored} & =\frac{E_{\text{stored\_rate}}}{P_{\text{pv}}}\\ & =\eta_{\text{ctrl}}\cdot \eta_{\text{batt}} \end{array}$$ (16)

These relationships allow for the quantitative evaluation of the battery’s effective charge rate and overall system energy conversion efficiency under varying solar irradiance conditions.

## Experiment and simulation

### Overview of system setup

**Fig 3:**
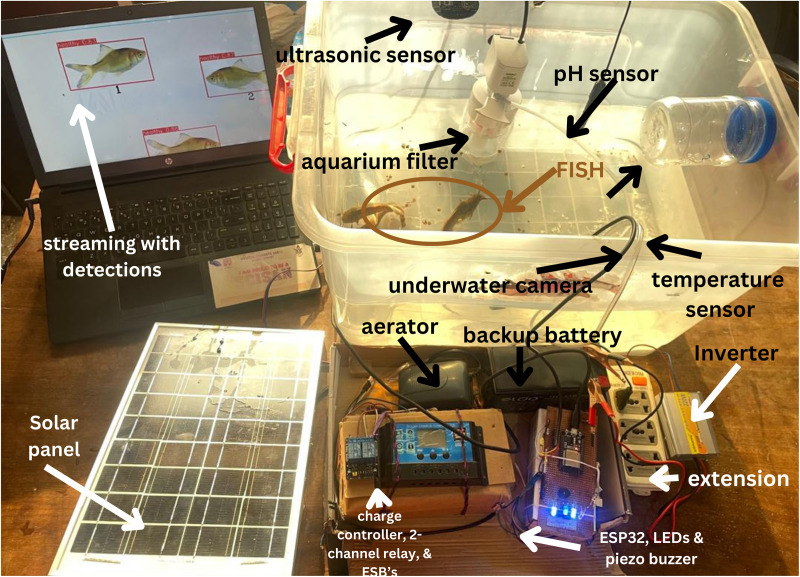
System Overview.

**Fig 4:**
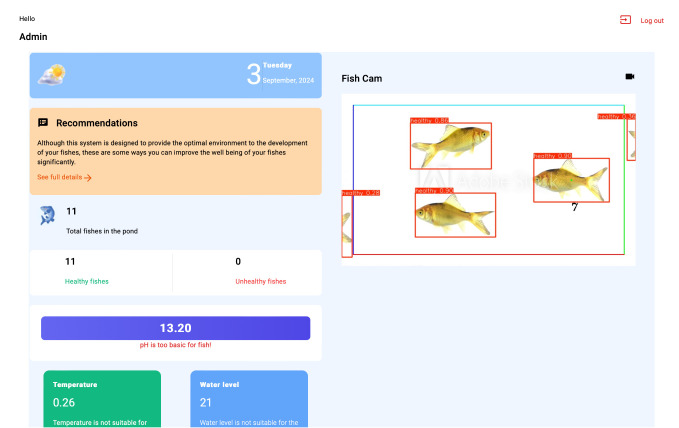
Angular dashboard showing values of water parameters and live detections.

Fig 3 and Fig 4 shows the complete setup of the sensors for monitoring water parameters and a typical display of parameters on the dashboard of the system respectively. The sensors are strategically installed to ensure accurate measurement of water quality. The feed container is mounted on a servo motor at the top of the container. When activated, the motor rotates at a predefined angle to dispense feed in controlled quantities. All sensors and actuators operate continuously. An ultrasonic sensor is positioned at the top of the container to measure water levels. When critical thresholds for water level, pH, or temperature are exceeded, an LED and a piezo buzzer trigger an alert to notify the user. The system is setup such that in any event of use, a fish can move freely in the water without any interference from system components. The aerator and aquarium filter are continuously powered to ensure adequate oxygen levels and maintain water quality by removing waste. Two Energy Storage Batteries(ESBs) are connected in parallel and covered in a cardboard package to shield them from water exposure. A charge controller with a two-channel relay is integrated into the system for efficient power management. The solar panel’s output is fed directly into the charge controller to charge the batteries. Additionally, a backup ESB is included to extend the system’s operational time.

### Machine learning for fish detection and disease diagnosis

**Fig 5:**
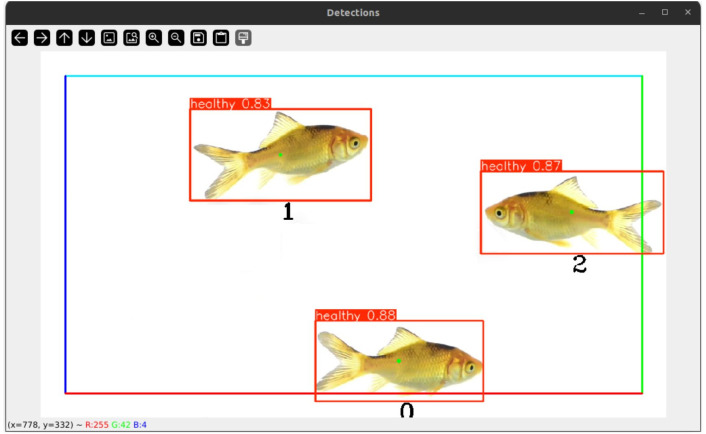
Detections. (a) Bacterial gill disease (b) Saprolegniasis (c) Red gill disease (d) White tail disease.

**Fig 6:**
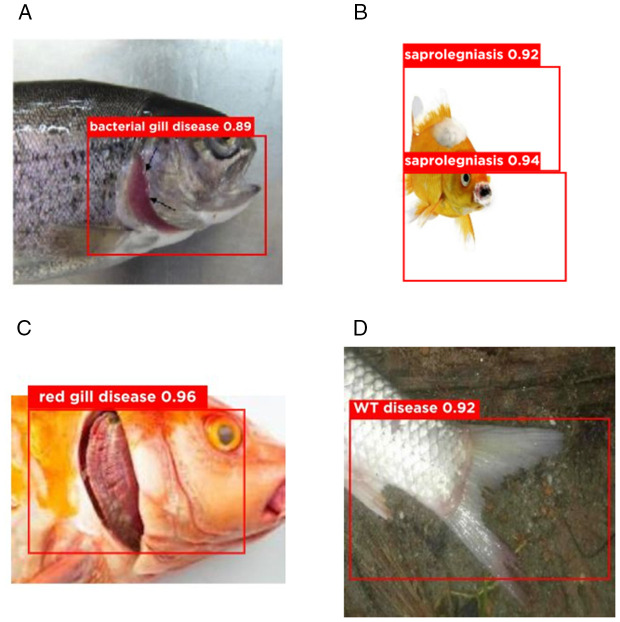
Results illustrating the performance of the trained model in detecting fish diseases across multiple classes.

We developed a mobile application using Android Studio and a web application using Angular, ensuring easy access regardless of mobile architecture differences. The YOLOv5s pre-trained model was fine-tuned to detect fish and diagnose diseases. The machine learning model was integrated into a user-friendly system, allowing users to monitor fish remotely from any location. Users can pause, stop, and resume live streaming in the fish tank as needed. The model detected fish by drawing bounding boxes around them and labeling their health status and xy coordinates where fish is found in the image. We defined a tracker class that effectively tracked fish movement by plotting a dot at the center of each fish within its bounding box. Each detection was assigned a unique ID for tracking and counting. When a fish’s center point crossed the frame’s border, the counter automatically incremented. Fig 5 and Fig 6 illustrates the detection results, displaying the bounding box, confidence score, status, and ID for each fish.

### Solar energy and ESB integration

Fig 3 illustrates a setup of the system’s connections. The solar panel is linked to a charge controller, which charges two lithium-ion batteries connected in series. The charge controller’s battery output is connected to a 2-channel relay, which regulates the charging process. Each relay channel is wired in series with the positive terminal of each battery. To power the system, a 12V DC to AC inverter converts the battery output to AC, ensuring all primary components can draw power. The system architecture is categorized into primary and secondary components. Primary components rely on AC power and can supply power to secondary components. The primary components include the Raspberry Pi, and the inverter, whereas the secondary components include the ESP32 module, oxygen pump, aquarium filter, etc. Secondary components are powered by the primary components. A serial communication link was established between the Raspberry Pi and ESP32 module, with the Raspberry Pi supplying power to the ESP32.

**Fig 7:**
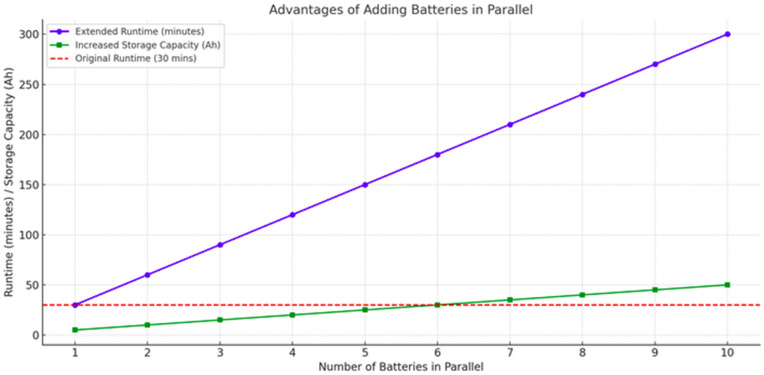
Simulation showing the direct proportionality between number of ESBs and Total Duration of 30 minutes.

To ensure a constant power supply, we conducted a simulation to determine the required number of batteries. Results showed a direct proportionality between the number of batteries and the total operational time of the system. Fig 7 presents the impact of adding batteries in parallel on both runtime and storage capacity: The runtime(blue line) shows how the runtime increases linearly as the number of batteries in parallel increases. For example, with 2 batteries, the runtime doubles to 60 minutes. With 3 batteries, it triples to 90 minutes, and so on. Storage Capacity (green line) shows the total storage capacity, which also increases linearly with the number of batteries. The original runtime(red dashed line) marks the runtime of 30 minutes with a single battery, providing a reference point. Adding batteries in parallel effectively increases the total capacity and runtime, making the system more robust for longer operational periods.

## Result and discussions

**Fig 8:**
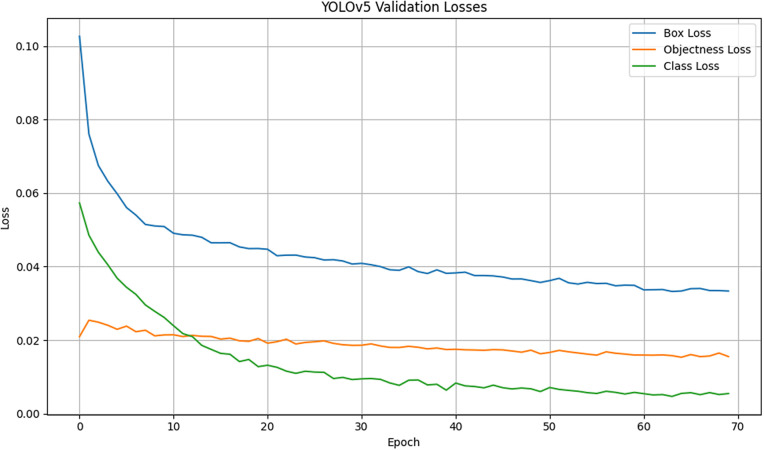
Training Losses.

Green fish farming has gained much research attention to boost fish production, minimize waste and pollution, and reduce the fish mortality rate. However, in practice, green fish farming is costly and tedious as it demands an intensive labor force, technical know-how, and a high cost of electricity in some cases. Locally, green fish farming is often done on a small scale with sustainability challenges. Most people in the fish business would rather go into commercial fishing that relies on harvesting wild fish instead of the cost-effective green fish farming methods available. In this paper, we present low-cost green fish farming equipment designed to automate standard labour-intensive fish farming processes such as fish feeding Fig 3). The proposed approach leveraged computer vision technology and renewable energy sources (Solar energy) to increase fish productivity and contribute to food security, making it more cost-effective and eliminating the need for intensive labor, whilst maintaining a reliable and efficient supply of energy by using bifacial solar panels combined with more durable and powerful energy storage battery solutions, like lithium-ion batteries, which offer greater energy density and longer life cycles. We evaluate the performance of our model during and after training. Certain key performance metrics, like recall, F1-score, precision, and loss trends, were used. Visualizations of the performance of the model are shown in:

Fig 8 shows the training losses of the fish disease detection model of 50 epochs. These include:

1. **Box Loss (blue)**, which reflects inaccuracies in estimating the bounding boxes of identified objects;
2. **Objectness Loss (orange)**, which measures how confidently the model predicts the presence of an object within a proposed bounding box; and
3. **Class Loss (green)**, which evaluates how accurately the model classifies the detected objects.

From Fig 8 we can notice that as training progresses, the losses begin to stabilize, suggesting the model has converged and further training offers limited benefits.

**Fig 9:**
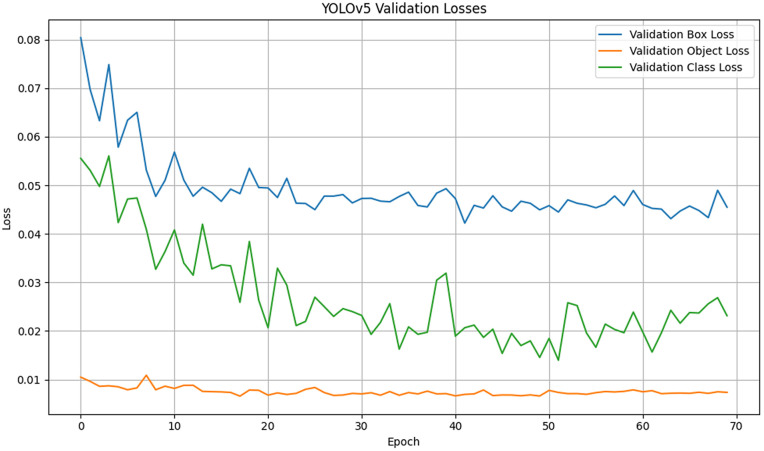
Validation Losses.

The validation losses of the fish disease detection model over 50 epochs is shown in Fig 9. Validation Box Loss(Blue) represents errors in predicting bounding boxes for the validation dataset. It swings intensively with the first epochs, but gradually stabilizes with more epochs, suggesting stability in localization predictions. Validation Object Loss(orange) measures the model’s confidence in correctly identifying the presence of objects within predicted regions. Validation losses exceed training losses, as is expected, since the model is tested on previously unseen data. Stabilization in later epochs suggests the model is not overfitting. Small fluctuations in validation losses indicate noise or challenging samples in the validation set.

**Fig 10:**
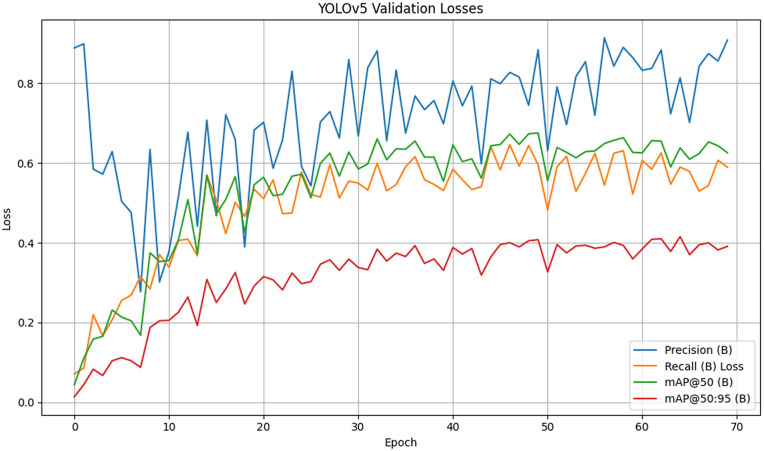
Performance Metrics.

Fig 10 shows the overall performance of the model over 50 epochs. Precision steadily improves, stabilizing at 0.86, indicating strong accuracy in positive detections. Recall rises on the start, but stabilizes at 0.70, suggesting the model misses some true positives. mAP@50 stabilizes at approximately 0.73, demonstrating good performance in simpler detection scenarios. However, mAP@50:95 stabilizes at a lower value of 0.60, reflecting challenges in accurate localization for more complex cases. The model illustrates strong precision and excels in simpler detection, but struggles with recall and localization in difficult scenarios, showing stable performance throughout training.

**Fig 11:**
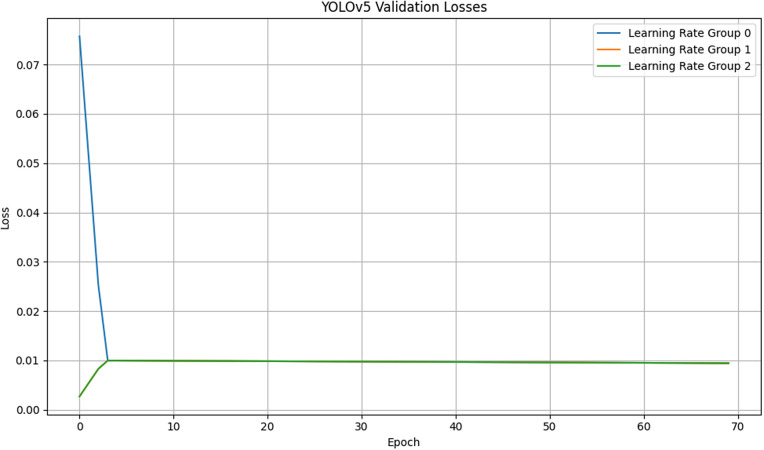
Learning Rate Per Group.

Fig 11 shows how the model’s learning rate progresses during training. Learning Rate Group 1 (orange) and 2 (green) have identical trajectories that converge early on. They start at 0.0027, gradually increase to 0.01, and then stay constant for the remainder of the training period. This initial increase enables the model to respond effectively to the data while avoiding significant, unstable parameter adjustments. Meanwhile, Learning Rate Group0 peaks at 0.0757 before steadily falling until it reaches Group2’s level of 0.01, where it remains constant. This controlled depreciation promotes steady and effective convergence.

### Summary of model performance metrics across selected epochs

Table 1 presents a summarized overview of the model’s performance across 50 epochs. The metrics shown include precision, recall, mean Average Precision at IoU threshold 0.5 (mAP@0.5), and mean Average Precision across multiple IoU thresholds ranging from 0.5 to 0.95 (mAP@0.5:0.95). The precision and recall values indicate the model’s ability to correctly identify relevant instances, while the mAP metrics provide a comprehensive evaluation of the model’s detection accuracy across different thresholds. An overall increasing trend is observed across the metrics, suggesting that the model improves its detection capabilities as training progresses.

**Table 1:** Model metrics for 50 epochs.

Epochs	metrics_precision	metrics_recall	metrics_mAP_0.5	metrics_mAP_0.5:0.95
10	0.6723	0.5328	0.5521	0.3247
20	0.7235	0.5734	0.5928	0.4129
30	0.7689	0.6137	0.6412	0.4835
40	0.8176	0.6624	0.6831	0.5567
50	0.8594	0.7015	0.7258	0.6023

## Conclusion

The shift toward solar-powered automated fish monitoring systems has set the stage for more sustainable aquaculture. While this system integrates solar energy, computer vision, and environmental sensors to improve fish farming practices, minor challenges such like sensor inaccuracies could be improved. Future iterations must address this issue through improved sensor quality. With continued innovation, this technology has the potential to revolutionize aquaculture by making it more efficient, autonomous, and environmentally friendly, ensuring long-term sustainability for rural and coastal communities.

In this paper, we have designed a practical system architecture that enables the development of a solar-based automated green fish farming system that is cost-effective. The system works along with an AI-based software that fundamentally enables fish monitoring, disease detection and waste management. Results obtained from our experimental simulations depicts that the proposed system is efficient, reduces operational cost, reduces fish mortality rate and can boost fish productivity.
